# In-Situ Synchrotron HEXRD Study on the Micro-Stress Evolution Behavior of a Superalloy during Room-Temperature Compression

**DOI:** 10.3390/ma16103761

**Published:** 2023-05-16

**Authors:** Hao Wang, Ruolan Tong, Guangxu Liu, Aixue Sha, Lin Song, Tiebang Zhang

**Affiliations:** 1 Beijing Institute of Aeronautical Materials, AECC, Beijing 100095, China; 2 State Key Laboratory of Solidification Processing, Northwestern Polytechnical University, Xi’an 710072, China

**Keywords:** FGH96 superalloy, in situ synchrotron HEXRD, load partitioning, room-temperature compression

## Abstract

The residual stress generated during heat treatment of nickel-base superalloys will affect their service performance and introduce primary cracks. In a component with high residual stress, a tiny amount of plastic deformation at room temperature can release the stress to a certain extent. However, the stress-releasing mechanism is still unclear. In the present study, the micro-mechanical behavior of the FGH96 nickel-base superalloy during room temperature compression was studied using in situ synchrotron radiation high-energy X-ray diffraction. The in situ evolution of the lattice strain was observed during deformation. The stress distribution mechanism of grains and phases with different orientations was clarified. The results show that at the elastic deformation stage, the (200) lattice plane of γ′ phase bears more stress after the stress reaches 900 MPa. When the stress exceeds 1160 MPa, the load is redistributed to the grains with their <200> crystal directions aligned with the loading direction. After yielding, the γ′ phase still bears the main stress.

## 1. Introduction

Nickel-based superalloys are widely used in aerospace and aeronautical industry for their high strength, excellent creep resistance, fatigue resistance, and corrosion resistance [1,2]. Powder metallurgy (PM) nickel-based superalloys with a high volume fraction of γ′ strengthening phase are used for the turbine disks on aero-engines. However, in the large-scale components, the uneven temperature distribution during heat treatment causes significant residual stress, which has a significant impact on the dimensional stability of disks during the subsequent machining and service process. Therefore, the distribution and evolution mechanisms of internal stress in PM superalloy components have always been a concern of researchers.

There are three types of residual stress [3]. Type I is macro residual stress, which is caused by uneven plastic deformation within the whole part. Type II is intergranular residual stress and type III is intragranular residual stress, both of which are micro-stresses. They are related to the deformation incompatibility between grains and lattice defects (vacancies, interstitial atoms, dislocations, etc.). PM nickel-based superalloy components usually contain a high level of residual stress after forging and heat treatment [4,5,6], which has a negative effect on the dimension stability and fatigue life of the component. However, pre-deformation could improve fatigue performance of the materials. Through the evolution of different types of texture in the pre-deformed Al-Cu-Li alloy, the strong Gaussian texture aided the reduction of the fatigue crack growth rate and increased the damage tolerance [7]. In magnesium alloy AZ31, {10$\overset{-}{1}$2} twins were introduced by pre-compression deformation, which improved the fatigue performance of the sample [8]. The residual compressive stress increased the effective fatigue threshold, inhibited crack propagation, and prolonged the fatigue life of the material [9]. Since annealing (which will introduce uneven cooling as well) cannot effectively lower the residual stresses inside the γ/γ′ alloy turbine disk, pre-spinning is usually used in industry to release and redistribute residual stress in order to obtain a better service stability and durability. It was found that the micro plastic deformation during pre-spinning can effectively lower residual stresses of the turbine disk [10], which is due to the internal stress evolution caused by the variation of interplanar spacing during deformation. Therefore, it is necessary to accurately measure the micro stress and strain during cold deformation.

In addition to neutron diffraction, synchrotron X-ray diffraction is a reliable method for accurately measuring the interplanar spacing. In particular, synchrotron radiation high-energy X-ray diffraction has a higher angular resolution, which has irreplaceable advantages in accurate determination of cell parameters. Coakley et al. [11] measured the lattice strain evolution during tensile tests of a polycrystalline nickel-base superalloy and found that the strain transferred to {220}γ after initial yielding of {200}. In the meantime, the strain was transferred from γ phase to γ′ phase. The two phases deformed together afterwards. Ma et al. [12] evaluated the load distribution of grains/phases with different orientations using in situ loading of a superalloy and obtained the critical shear stress of each phase. Goodfellow et al. [13] found that the stress had an intergranular distribution under low loads and an interphase distribution under high loads. The large lattice misfit resulted in the initiation of interphase load distribution under low stress. Prasad et al. [14] analyzed the diffraction data of six {hkl} planes during tensile deformation and found that the dislocation density in the additive-manufactured crack-free Hastelloy was higher than that in the forged alloy. The proportion of screw dislocation was higher. Stress distribution behavior is closely related to the microstructures and volume fraction of γ′ strengthening phases, which is of great significance for revealing the deformation mechanism of precipitation strengthening superalloys. However, the relevant reports are still insufficient.

In this paper, compression experiments of a PM nickel-base superalloy at room temperature were carried out. The behavior of internal stress accumulation and stress distribution during micro plastic deformation was revealed by in situ synchrotron radiation X-ray diffraction. This work can offer a useful reference for improving the dimension stability and fatigue life of superalloy turbine disk by revealing the internal stress distribution.

## 2. Experiment

**Figure 1:**
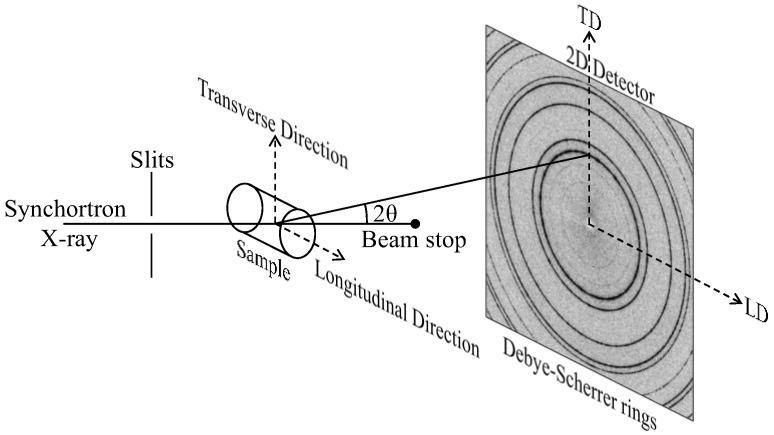
Schematic diagram of the in-situ compression HEXRD experimental device.

The FGH96 superalloy billet was prepared through the PM method. The chemical composition (in wt. %) of FGH96 superalloy is shown in Table 1. The FGH96 alloy powders (<270 mesh) were consolidated by hot isostatic pressing (HIP) at 1170 °C and a stress condition of 140 MPa/5 h. The HIPed ingot was then hot extruded and isothermal forged at subsolvus temperatures. After forging, the pancake was heat treated at 1160 °C for 4 h for solid solution treatment, and then aged at 760 °C for 16 h, followed by air cooling. The microstructure of the aged ingot was observed in the optical microscope and scanning electron microscope (SEM). Samples were prepared by grinding and chemical polishing. Cylinders with a diameter of 4 mm and a height of 8 mm were machined from the ingot for in situ compression tests. In situ high energy synchrotron X-ray diffraction (HEXRD) measurement was conducted in the Deutsches Elektronen-Synchrotron (DESY) in Hamburg, utilizing the P07EH3 beamline of Helmholtz- Zentrum Geesthacht at PETRA III, with a beam size of 0.4 × 0.4 mm^2^ and a beam energy of 100 KeV (wave length 0.124 Å). The schematic diagram of the experimental device is shown in Figure 1. The longitudinal direction (LD, φ = 0°) is parallel to the load axis and cylinder axis. The transverse direction (TD, φ = 90°) is perpendicular to the load axis. At room temperature, the cylinder was compressed, with a strain rate of 10^−3^ s^−1^ and unloaded at 5.4% strain.

The diffraction images were recorded on a two-dimensional detector. Debye–Scherrer diffraction rings were unrolled by FIT2D software and integrated along the LD/TD within the range of ±10° into a series of one-dimensional XRD patterns. Matlab software was used to fit diffraction peaks according to the pseudo-voigt function, which also calculated the peak position, full width at half maximum (FWHM), and peak intensity of each {hkl} reflection. The corresponding interplanar spacing $d_{hkl}$ was calculated according to Bragg’s Law:

(1) $$d_{hkl}\;=\;\lambda /2\sin\theta_{hkl}$$

where λ (0.124 Å) is the wavelength and 2$\theta_{hkl}$ is the diffraction angle of the X-ray. The lattice strain ε_hkl_ was then calculated as:

(2) $$\varepsilon_{\mathrm{hkl}}=(d_{hkl}^{1}-d_{hkl}^{0})/d_{hkl}^{0}$$

where $d_{hkl}^{0}$ and $d_{hkl}^{1}$ are the interplanar spacing *d* of the {hkl} lattice planes in the conditions of initial stress-free state and loading state, respectively.

The very similar lattice constants of γ and γ′ phases generated overlapped diffraction peaks, which made it difficult to determine the peak positions and lattice misfit. The lattice spacing *d_hkl_* of superlattice reflections was used to derive the parallel high exponential fundamental {2h2k2l} reflections. The positions of γ-{2h2k2l} reflections were obtained by fixing the position of γ′-{2h2k2l} reflections and deconvolution. In order to ensure the reliability of the fitting process, the integral intensities of γ and γ’ peaks, which are proportional to the volume fractions of the corresponding phases, were considered as constants, according to the following equation [12]:

(3) $$\frac{I_{2h2k2l}^{\gamma \prime }}{I_{2h2k2l}^{\gamma }}=\frac{{{|F}_{2h2k2l}^{\gamma \prime }|}^{2}}{{\left|F_{2h2k2l}^{\gamma }\right|}^{2}}\frac{v_{f}}{\left(1-v_{f}\right)}$$

where *I_hkl_* is the intensity of {hkl} peak and *v_f_* is the volume fraction of γ′ phase, measured as 0.4. *F_hkl_* is the structural factor determined by Equation (4):

(4) $$F_{hkl}=b_{c}e^{2\pi i(0h+0k+0l)}+b_{f}(e^{2\pi i\left(0.5h+0.5k+0l\right)}+e^{2\pi i\left(0.5h+0k+0.5l\right)}+e^{2\pi i\left(0h+0.5k+0.5l\right)})$$

where *b_c_* and *b_f_* are the scattering lengths of corner atoms (primarily Al atoms) and face atoms (primarily Ni atoms), which are 0.35 × 10^−12^ cm and 1.03 × 10^−12^ cm, respectively. For the γ phase, it is assumed that *b_c_ = b_f_* = *b_Ni_*. The value of lattice misfit was calculated as:

(5) $$\delta =\frac{2(a_{\gamma^{'}}-a_{\gamma })}{a_{\gamma^{'}}+a_{\gamma }}$$

where $a_{\gamma }$ and $a_{\gamma^{'}}$ are the lattice constants of the γ and γ′ phases, respectively.

## 3. Results

### 3.1. Microstructure and Compression Curves of FGH 96 Superalloy

**Figure 2:**
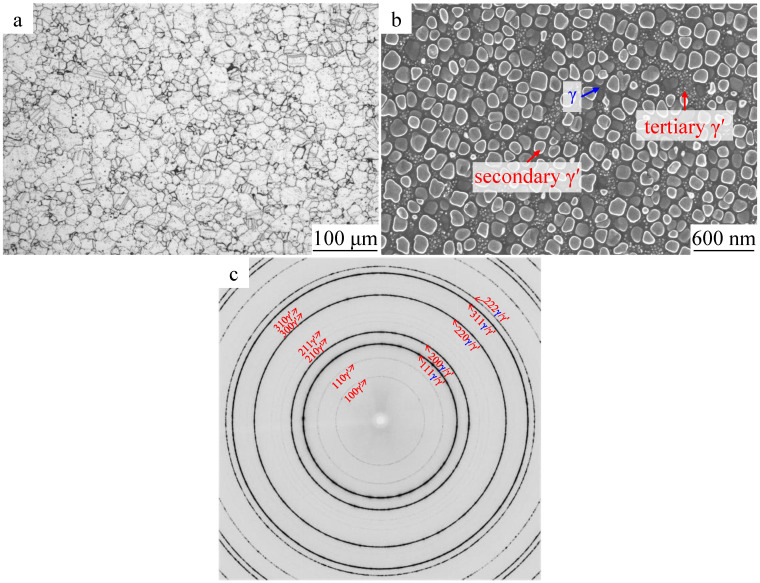
(**a**) Optical image of FGH 96 alloy after forging; (**b**) backscattered electron SEM image of the secondary and tertiary γ′ precipitates; (**c**) synchrotron radiation diffraction pattern of the microstructure before deformation.

The microstructure of the superalloy sample used for in situ compression tests is shown in Figure 2a,b, which displays the overall grain statistics observed by an optical microscope and the distribution of γ′ precipitates observed by SEM. Figure 2a shows a fine-grain microstructure with an average grain size of approximately 30 μm. Two kinds of γ′ precipitate exist—namely, the secondary γ′ precipitates nucleated during cooling after solution and tertiary γ′ precipitates formed and grown during the aging treatment. However, there was no primary γ′ precipitates phase located at the grain boundary. The volume fraction of γ′ precipitates was approximately 40%. Figure 2c is the diffraction pattern obtained by synchrotron radiation measured before the compression. Since a number of peaks of the L1_2_-γ′ phase are overlapped with the peaks of fcc-γ, most diffraction rings are labeled as the common reflections of γ′/γ. However, a few superlattice diffractions, such as (100)_γ′_ and (110)_γ′_, can also be clearly seen on the pattern. Some other peaks exhibit higher intensity, for example (111) and (200), owing to the overlap effect of γ and γ′ phases. The fine grain size of the microstructure can also be reflected by the integrity of the diffraction rings in Figure 2c, indicating a good homogeneity of the microstructure.

**Figure 3:**
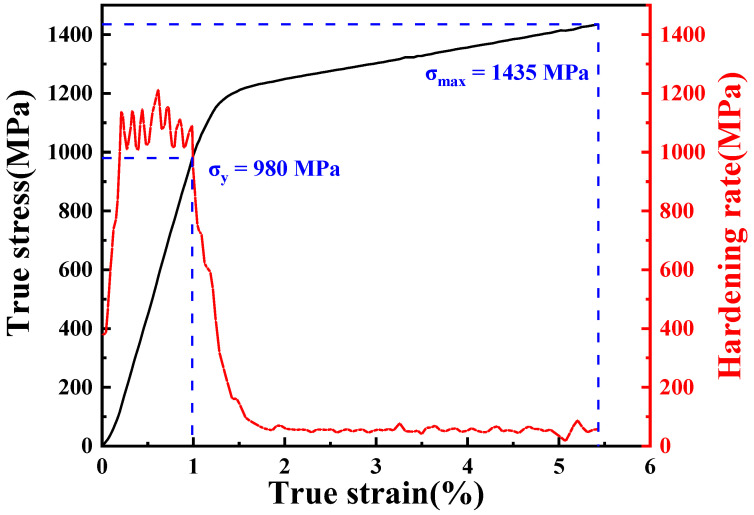
True stress-true strain curve obtained in the in situ compression measurement at room temperature.

The true stress-strain curve of FGH96 superalloy obtained by in situ synchrotron radiation compression is shown in Figure 3. The stress increased rapidly in the elastic stage, while a specific yield point is difficult to obtain. The red curve in Figure 3 shows the evolution of the work hardening rate. When the strain reached 1%, the work hardening rate rapidly decreased, and the sample yielded at about 980 MPa. After that, the strain increased rapidly with the increase in stress while the work hardening rate almost remained constant during the plastic deformation. The maximum true strain was 5.4% and the corresponding true stress was 1435 MPa.

### 3.2. Evolution of the Reflections during the In-Situ Compression Test

**Figure 4:**
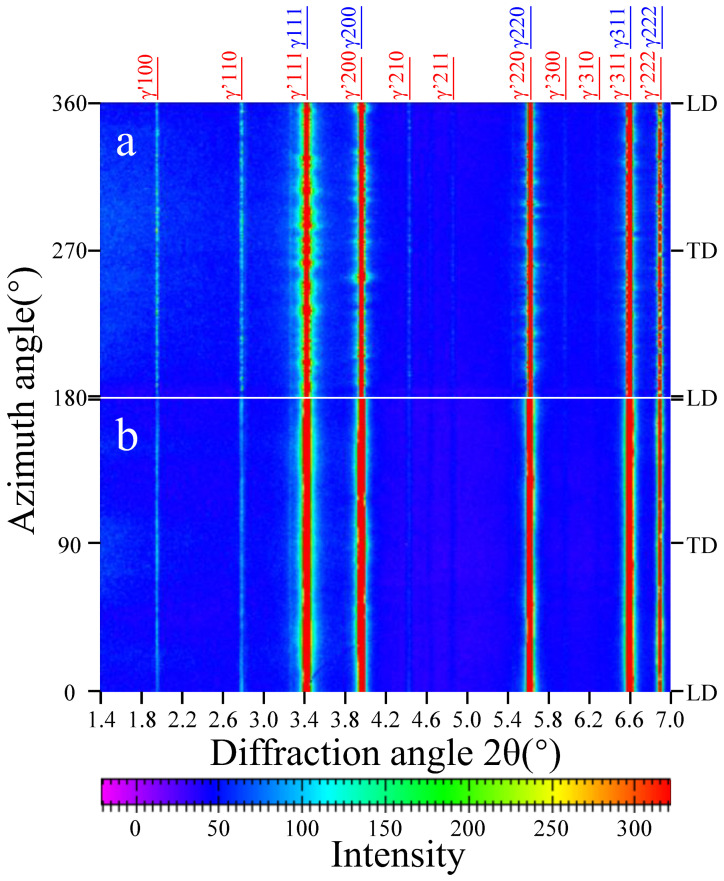
Unrolled two-dimensional diffraction rings of the sample (**a**) before and (**b**) after compression at room temperature.

The unrolled two-dimensional X-ray diffraction patterns of the alloy before and after compression are shown in Figure 4a,b, respectively. The reflections of (100), (110), (210), and (211) planes are superlattice diffractions, which correspond to the γ′ phase. The intensity of (210)_γ′_ and (211)_γ′_ diffraction peaks decreased after deformation. The intensity of (111), (200), (220), (311), and (222) diffraction peaks was much higher than the other lattice planes, indicating that the lattice constants of the γ and γ′ phases are almost the same. According to the superlattice diffraction pattern of the γ′ phase and the peak separation processing, the lattice constants of the γ and γ′ phase were determined *a_γ_* = 3.587 ± 0.005Å and *a_γ’_* = 3.589 ± 0.005Å, respectively. Thus, the lattice misfit was 0.056 ± 0.005%. Comparing Figure 4a,b, the width of these diffraction peaks increased significantly after deformation, indicating that plastic deformation caused a large residual strain. It should be noted that the phase constitution (the volume fractions of γ and γ′ phases) had not changed before and after deformation.

**Figure 5:**
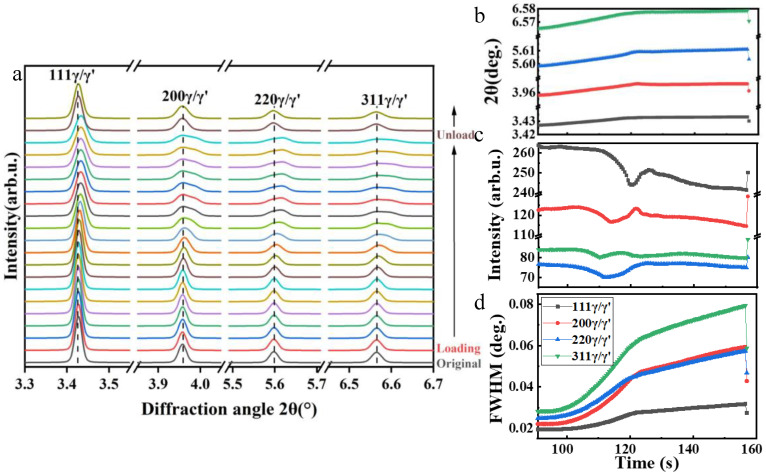
Evolution of one-dimensional diffraction pattern during compression: (**a**) overall one-dimensional HEXRD curve; (**b**) offset 2θ value; (**c**) peak intensity; (**d**) FWHM.

Figure 5a shows the evolution of the diffraction peaks during room temperature compression. During loading, an obvious deviation of the peak position from the normal value can be observed. After unloading, the diffraction peaks almost returned to being symmetrical, but the full width at half maximum (FWHM) of the peaks did not return to the original state (Figure 5d). In addition, the 2θ values of peaks also shifted (Figure 5b). Due to the effect of residual stress caused by the plastic strain, the interplanar spacings decreased and the diffraction peaks broadened, which were obviously caused by dislocation movement. A certain number of dislocations made the interplanar spacing of the slip planes unable to fully recover to the initial state, that is, a certain amount of the lattice stress caused by the absence of semi atom plane of edge dislocations remained in the microstructure [15]. Elastic distortion appeared around the dislocation line. When a large number of dislocations were activated, the accumulated lattice distortion caused the interplanar spacing to deviate from the normal value, and the FWHM increased at the same time. During compression, the shape and position of the diffraction peaks changed significantly, and the asymmetry gradually increased, which is probably due to the increase in the γ/γ′ misfit. Specifically, the diffraction peak intensity, 2θ angle, and the FWHM data obtained by fitting in Figure 5b–d are analyzed as follows.

In the elastic deformation stage, with the increase in stress, the lattice plane spacings decreased while the 2θ angle increased. Meanwhile, the peak position gradually shifted to the right, as shown in Figure 5b. The shift of (220) and (311) diffraction peaks was the largest while the shift of (111) diffraction peaks was relatively small. During the stable plastic deformation stage, the position of the diffraction peak changed gradually, which showed that the influence caused by elastic distortion is increasingly apparent. After unloading, the values of 2θ angle decreased rapidly, whereas it was still higher than that before the deformation. Generally, the diffraction peak intensity is related to the preferred orientation. In Figure 5c, the diffraction intensity decreased at the elastic deformation stage, whereas it fluctuated at the beginning of the plastic deformation, and then continued to decrease slowly at the stable plastic deformation stage. Specifically, the intensity of (111) diffraction peak decreased significantly after unloading while the intensity of other diffraction peaks increased. Due to the different deformation ability of γ and γ′ phases, the asymmetry of diffraction peaks increased rapidly and even double peaks appeared. Therefore, single-peak fitting cannot fully satisfy the practical situation. Thus, further separation of single γ and γ′ diffraction peaks is necessary, which will be discussed in Section 4.2.

The evolution of FWHM with time during compression is shown in Figure 5d. After plastic deformation, the FWHM values of (111), (200), (220), and (311) planes increased by 21%, 44%, 36%, and 49%, respectively, indicating the dislocation density in the alloy increased rapidly and the defects accumulated continuously [16]. The difference of the widening rate of each lattice plane was obviously caused by the anisotropy of the crystal structure. As the dislocations were fully located on the close-packed lattice plane (111) with the largest slip distance, the increase in FWHM of the (111) plane was not as significant as the other planes. Since the dislocations were partially located on other lattice planes, the misfit degree of the atoms on the adjacent lattice planes was higher than that on the glide plane, resulting in a faster widening rate of the peaks. In addition, the increase in the peak width of (311) lattice plane was the largest, suggesting that the dislocation slip had the strongest influence on its interplanar spacing. This phenomenon can be used as a basis for analyzing the deformation degree of cubic structures.

## 4. Discussion

### 4.1. Intergranular Microstress Evolution

**Figure 6:**
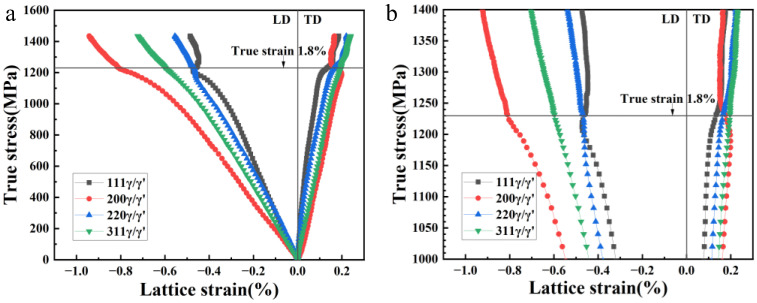
True stress-lattice strain curve during room temperature compression: (**a**) overall evolution of each lattice strain with true stress in the whole deformation process; (**b**) evolution of each lattice strain above 1000 MPa.

The true stress-lattice strain evolution during compression is shown in Figure 6a. The lattice strain value along the longitudinal direction (LD) was negative. For the convenience of the discussion, the absolute value will be considered in the following part. The deformation process can be divided into three stages: the elastic deformation stage, the elasto-plastic transition stage, and the stable plastic deformation stage. In the first stage, the linear response between the lattice strain and true stress was observed for the (111), (200), (220), and (311) planes. Due to the strong anisotropy of the γ′ phase, the <200> direction was the most likely to generate elastic strain, while the <111> direction had the largest stiffness, meaning that its strain was smaller under the same stress conditions. The elastic stiffness of <220> and <311> directions was moderate, which is consistent with the previous results [13,17]. At the stress level of 900 MPa, the (111) reflection exhibited a downward deviation first and other lattice planes followed immediately, whereas they still remained in the elastic deformation stage.

In the second stage, the (220) reflection firstly yielded at 1160 MPa and then the (111) reflection yielded at 1200 MPa. The gradient of the curve changed dramatically in this period. Generally, the (111) reflection yielded first because it was subjected to the maximum stress. However, due to the effect of plastic anisotropy, the Schmidt factor for the a/2<110> slip in the grains with their (100) and (110) planes perpendicular to the LD was much larger than those with their (111) planes perpendicular to the LD. Therefore, the latter ones yielded later and bore the transferred load [18]. Figure 6b shows the enlarged view of the lattice plane evolution above 1000 MP. After the yielding of (220) and (111) planes, the (200) plane bore a higher load and the lattice strain increased to a more negative value, showing a downward offset of the (200) curve. The behavior of the (311) reflection always remained close to linear, which was consistent with previous studies [19,20,21].

The third stage started at 1230 MPa. As shown in Figure 6b, an inflection point appeared in each lattice strain, and the corresponding true strain at this moment was 1.8%. The upward deviation of the curve indicated that obvious plastic deformation occurred. The lattice strain of (111) reflection along the LD direction and the (200) reflection along the transverse direction (TD) showed an obvious decrease. This phenomenon might be due to the fact that the slip systems of the grains in favorable orientations were activated [22]. The dislocations began to slip, and part of the internal stress was released, resulting in lattice strain reduction. Taking the (111) lattice plane as an example, it can be seen from the detour of the strain curve that the absolute value of the lattice strain was about 0.5% when the strain reached 5.4%, which was slightly higher than the absolute value of the maximum elastic strain. This indicates that although the internal stress of the sample decreases for a while at the occurrence of plastic deformation, it soon shifts to an increasing trend. From the perspective of the deformation mechanism, the strain releasing effect caused by dislocation slip only existed at the initial stage of plastic deformation. The subsequent rise of the lattice strain indicated that dislocations were re-blocked and pinned after a certain distance of gliding, which corresponds to the macroscopical work hardening. This is also reflected in the compression stress-strain curve, where obvious work hardening occurs after yielding (Figure 3). Considering the microstructure of the alloy, this is due to the pinning effect of secondary and tertiary γ′ particles. Dislocations were blocked at the γ/γ′ phase interface and were thus unable to propagate for a long distance. Only when the load continued to rise to make the dislocations trapped at the interface slip into the interior of the γ′ phase could the strain be relaxed again. In addition, when the strain was 0.44%, the absolute value of the γ/γ′ lattice strain reached the minimum, which indicated that the lattice strain had been reduced to a lower level by micro-plastic deformation (dislocation slip). The corresponding engineering strain was 2.9%. It can be considered that if the sample was unloaded at this moment, the internal stress in the sample would be released to some extent.

### 4.2. Interphase Microstress Evolution

**Figure 7:**
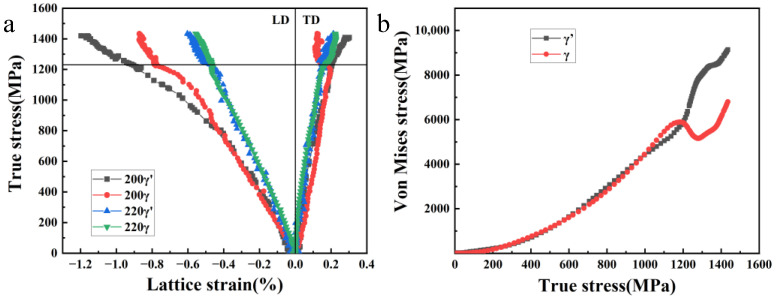
(**a**) True stress–lattice strain curves of (200) and (220) diffraction peaks of the γ and γ′ phases; (**b**) relationship between Von Mises equivalent stress and true stress of the γ and γ′ phases.

Although the diffraction peaks of γ and γ′ phases overlapped, different orientations of the phases would bear varying loads, which further complicated the deformation mechanism. Therefore, the diffraction peaks of the γ and γ′ phases should be discussed separately. Figure 7a shows the fitted true stress-lattice strain curves of the (200) and (220) diffraction peaks of γ and γ′ phases. Since the background has a great influence on the diffraction peaks of (100)_γ′_ and (110)_γ′_, the fitting results with a confident degree above 99.5% are selected. In the linear–elastic stage, the curves of the γ and γ′ phases almost coincided, indicating that γ and γ′ had a similar stiffness. As shown in Figure 7a, (220)_γ_/(220)_γ′_ yielded first and (200)_γ_/(200)_γ′_ bore higher stress, and, thus, the lattice strain with (200)_γ_/(200)_γ′_ orientation increased rapidly. When the stress reached 900 MPa, the γ phase in the direction of (200)_γ_//LD was more prone to plastic deformation than the γ′ phase, indicating that the dislocations were more likely to slip in the softer γ phase. At the same time, the γ′ phase bore a higher load and obvious interphase load redistribution occurred. On the (220) lattice plane, γ and γ′ phases had a nearly consistent deformation. After yielding, the γ phase shared the load with the γ′ phase, and the two phases deformed almost at the same strain rate. In the plastic stage, (200)_γ_ yielded first, resulting in an upward migration of true stress-lattice strain curve, while (200)_γ′_ bore a higher load and continued to deform. In the TD direction, (200)_γ_ yielded at 1200 MPa, and then its lattice strain decreased with the increase in stress, and the curve rebounded. It could be due to this that the dislocations slipped from γ to γ′ and then started to slip again, which redistributed the stress [12]. At the stable plastic deformation stage after 1230 MPa, the strain hardly increased and the stress–lattice strain gradient of (220)γ was large, indicating that more load was allocated to the γ′ phase.

Von Mises equivalent stress [23,24] can be used to observe the stress changes between γ and γ′ phases:

(6) $$\sigma_{VM}=\frac{1}{\sqrt{2}}\left[{\left(\sigma_{11}-\sigma_{22}\right)}^{2}+{\left(\sigma_{22}-\sigma_{33}\right)}^{2}+{\left(\sigma_{11}-\sigma_{33}\right)}^{2}\right]$$

(7) $$\sigma_{11}=\frac{E}{1+\vartheta }\varepsilon_{11}+\frac{\vartheta E}{\left(1+\vartheta )(1-2\vartheta \right)}\left(\varepsilon_{11}+\varepsilon_{22}+\varepsilon_{33}\right)$$

(8) $$\sigma_{22}=\sigma_{33}=\frac{E}{1+\vartheta }\varepsilon_{22}+\frac{\vartheta E}{\left(1+\vartheta )(1-2\vartheta \right)}\left(\varepsilon_{11}+\varepsilon_{22}+\varepsilon_{33}\right)$$

where $\sigma_{VM}$ is Von Mises effective stress. $\sigma_{11}$ and $\sigma_{22}$ are the principal stresses along the LD and TD, respectively. E is the elastic constant of diffraction, *ϑ* is Poisson’s ratio, $\varepsilon_{22}$ and $\varepsilon_{33}$ is the lattice strain for LD and normal direction (ND), respectively. $\varepsilon_{22}$ equals to $\varepsilon_{33}$.

Poisson’s ratio is the average value of the lattice strain ratio at TD and LD. The lattice plane (220) was selected to calculate the stress of γ′ and γ phases, as shown in Figure 7b. Although fitting error makes the curve fluctuate to some extent, it still reflects the evolution trend of load bearing on the γ and γ′ phases. During the elastic stage, there was almost no difference in the load distribution between the two phases. The equivalent stress in the two phases was concordant. When the stress reached 1200 MPa, the stress on the γ phase decreased and transferred to the γ′ phase. During the subsequent plastic deformation stage, the γ′ phase bore higher stress continuously.

## 5. Conclusions

The deformation and stress partitioning mechanisms of PM FGH96 nickel-based superalloy during small strain compression at room temperature were investigated using in situ HEXRD technique. It was found that the (111) lattice plane had the maximum stiffness while (200) had the minimum. Between the γ/γ′ grains, when the applied stress reached 1160 MPa, the (220) lattice plane yielded first, and then the load was redistributed to grains with the <200> crystal directions aligned with the loading direction. Between the γ and γ′ phase, when stress was above 900 MPa, the (200)_γ′_ bore the main stress; when the stress reached 1200 MPa, the γ phase yielded first, and the stress was allocated to the γ′ phase. When the engineering strain was 2.9%, the lattice strain of (111) reduced to 0.44%, so it can be considered that the large pre-existing internal stress of the superalloy turbine disk can be released if unloaded at this strain. 

## Figures and Tables

**Table 1:** Chemical composition (wt. %) of the FGH96 superalloy.

Co	Cr	Mo	W	Al	Ti	Nb	C	B	Zr	Ni
12.9	15.7	4.0	4.0	2.1	3.7	0.7	0.05	0.03	0.05	Balance

## Data Availability

Data will be available on request.
